# When small effect sizes become huge: Synaesthesia is linked to very large differences in cognition

**DOI:** 10.1177/03010066231218911

**Published:** 2023-12-06

**Authors:** Jamie Ward

**Affiliations:** University of Sussex, UK

**Keywords:** individual differences, effect sizes, multivariate, synaesthesia

## Abstract

The replication crisis has taught us to expect small-to-medium effects in psychological research. But this is based on effect sizes calculated over single variables. Mahalanobis *D*, the multivariate equivalent of Cohen's *d*, can enable very large group differences to emerge from a collection of small-to-medium effects (here, reanalysing multivariate datasets from synaesthetes and controls). The use of multivariate effect sizes is not a slight of hand but may instead be a truer reflection of the degree of psychological differences between people that has been largely underappreciated.

One of the main lessons learned from the replication crisis in psychology is that small-to-medium effect sizes are likely to be the norm; perhaps to the extent that any result that deviates from this is viewed with suspicion. Funder and Ozer (2019) suggested that: “A very large effect size… in the context of psychological research is likely to be a gross overestimate that will rarely be found in a large sample or in a replication.” In this *Short and Sweet*, we point out that a collection of small-to-medium effects can constitute a statistically very large effect. We illustrate this principle with regards to the phenomenon of synaesthesia, but we speculate that this may well hold true across a wide range of psychological group differences in the literature.

Synaesthesia is an extraordinary perceptual experience in which music may be coloured, words may have tastes, and numbers are a visuo-spatial landscape. Synaesthesia has been linked to a wide variety of individual differences in domains such as perception, intelligence, and personality. Effect sizes are typically in the “ordinary” small-to-medium range on these measures, so there is an apparent mismatch between the extraordinary experiences of synaesthetes and more modest differences on standardised measures (Chun & Hupe, 2016; Rothen & Meier, 2010). The standard metric in such research is Cohen's *d* which is the distance between the mean of two standard normal distributions in units of SD (standard deviation). Cohen (1988) gives the indicative values of *d* > 0.3 as small, *d* > 0.5 as medium, and *d* > 0.8 as large. Less well known to psychologists are the categories of very large (*d* > 1.2) and huge (*d* > 2).

Let us imagine that synaesthetes have *d* = 0.5 (medium) for a memory task and *d* = 0.3 (small) for a perception task. What is the overall group difference across both tasks? Is it the average (i.e., a small *d* = 0.4), the sum (i.e., a large *d* = 0.8), or another value? A little known and seldom used effect size termed Mahalanobis *D* gives the answer.

Mahalanobis *D* is a multivariate version of Cohen's *d* that gives an effect size on the same SD scale. The key insight is that it considers the degree of relationship between multiple tasks or measures (via their covariance). If two tasks measure the same thing, then the Mahalanobis *D* will be an average of the effect sizes (a meta-analysis effectively works like this). But if the measures are orthogonal (90°) to each other, then it becomes a Euclidean distance √ (0.5^2 ^+ 0.3^2^) = 0.58, and so on for all possible degrees of relationship. To give a prior example from the literature, gender differences in personality are small on singular dimensions (average Cohen's *d* = 0.44) but become huge (Mahalanobis *D* = 2.71) when considered collectively (Del Giudice et al., 2012). For further details on this metric, including an R function that implements it, see Del Giudice (2019), and the current code and results are online for curious readers (https://osf.io/bnt8u/).

For synaesthesia, we reanalysed three published datasets, applying bias-corrected Mahalanobis *D* due to small samples relative to the number of variables. Ward and Filiz (2020) report 33 measures including tests of perception, creativity and memory and questionnaires relating to sensory sensitivity, mental imagery, personality, amongst others. There were 101 synaesthetes and 100 controls and the absolute effect sizes ranged from *d* of 0.00 to 1.11, with a mean of 0.22. The Mahalanobis *D* was a very large 1.494. Rouw and Scholte (2016) report 14 measures of intelligence, personality, and social-emotional regulation in 89 synaesthetes and 107 controls. The absolute effect sizes ranged from *d* of 0.02 to 0.74, with a mean of 0.31. The Mahalanobis *D* was a large 0.806. Chun and Hupe (2016) report 18 measures on tests of creativity, intelligence, and personality from 29 synaesthetes and 36 controls. The absolute effect sizes ranged from *d* of 0.03 to 0.82, with a mean of 0.31. The Mahalanobis *D* was a large 1.138. When looked at this way the cognitive profile of synaesthesia appears more “extraordinary” than “ordinary.”

Of course, we do not know the ground-truth to determine whether such estimates are accurate and there is little existing literature exploring the limitations of this statistical measure. There are debates around whether different thresholds for small, medium, and large need to be set for multivariate measures. For example, Stevens (2002) recommended threshold values of Mahalanobis *D* (based on calculations of *D*^2^) of 0.5 (small), 0.71 (medium), and 1 (large). One possible resolution is to look for converging evidence from other measures. Chun and Hupe (2016) performed a MANOVA (multivariate ANOVA) on their data which also pointed towards a large group difference (synaesthetes versus controls). An alternative approach is to use machine learning predictive models. These produce univariate outcomes (group classifications) and univariate effect sizes from multivariate data. Using this approach, Ward and Filiz (2020) also found a large effect (Cohen's *d* = 1.208) similar to the calculated Mahalanobis *D* of 1.494 from the same data. We don’t know which estimate is more accurate but they derive from mathematically very different calculations and are both large or very large effects.

Although we have illustrated this principle with respect to synaesthesia, the implications are broad. Psychological research typically deals with multivariate datasets but researchers choose to present this as multiple univariate effects (e.g. Cohen's *d*). This has some benefits in terms of understanding the relative importance of different variables. However, it also creates an impression that psychology is a “science of small effects.” This is perhaps misleading and unhelpful. The use of multivariate effect sizes is not a slight of hand but may instead be a truer reflection of the degree of psychological differences between people that has been largely underappreciated.
